# Graph neural networks and cross-protocol analysis for detecting malicious IP addresses

**DOI:** 10.1007/s40747-022-00838-y

**Published:** 2022-09-14

**Authors:** Yonghong Huang, Joanna Negrete, John Wagener, Celeste Fralick, Armando Rodriguez, Eric Peterson, Adam Wosotowsky

**Affiliations:** 1 Hillsboro, OR 97229 USA; 2 Brentwood, CA 94513 USA; 3 West Lakeland, MN 55082 USA; 4 Lubbock, TX 79403 USA; 5 Elizabeth, CO 80107 USA; 6 Lilburn, GA 30047 USA

**Keywords:** Graph neural networks, Machine learning, IP reputation, Network security

## Abstract

An internet protocol (IP) address is the foundation of the Internet, allowing connectivity between people, servers, Internet of Things, and services across the globe. Knowing what is connecting to what and where connections are initiated is crucial to accurately assess a company’s or individual’s security posture. IP reputation assessment can be quite complex because of the numerous services that may be hosted on that IP address. For example, an IP might be serving millions of websites from millions of different companies like web hosting companies often do, or it could be a large email system sending and receiving emails for millions of independent entities. The heterogeneous nature of an IP address typically makes it challenging to interpret the security risk. To make matters worse, adversaries understand this complexity and leverage the ambiguous nature of the IP reputation to exploit further unsuspecting Internet users or devices connected to the Internet. In addition, traditional techniques like dirty-listing cannot react quickly enough to changes in the security climate, nor can they scale large enough to detect new exploits that may be created and disappear in minutes. In this paper, we introduce the use of cross-protocol analysis and graph neural networks (GNNs) in semi-supervised learning to address the speed and scalability of assessing IP reputation. In the cross-protocol supervised approach, we combine features from the web, email, and domain name system (DNS) protocols to identify ones which are the most useful in discriminating suspicious and benign IPs. In our second experiment, we leverage the most discriminant features and incorporate them into the graph as nodes’ features. We use GNNs to pass messages from node to node, propagating the signal to the neighbors while also gaining the benefit of having the originating nodes being influenced by neighboring nodes. Thanks to the relational graph structure we can use only a small portion of labeled data and train the algorithm in a semi-supervised approach. Our dataset represents real-world data that is sparse and only contain a small percentage of IPs with verified clean or suspicious labels but are connected. The experimental results demonstrate that the system can achieve $85.28\%$ accuracy in detecting malicious IP addresses at scale with only $5\%$ of labeled data.

## Introduction

Year after year, the Internet continues to see drastic growth in new users. It is reported that the number of users has grown from 3.65 billion in 2018 to 4.66 billion in January of 2021 [1]. Not only are there more users, but there are significantly more devices connecting. Smartphones, refrigerators, home security cameras, and even light bulbs contribute to the growing Internet of Things network and are now fully connected to the Internet and consuming Internet protocol (IP) addresses. Unfortunately, many of these devices are not designed with cybersecurity as a priority and hence, are vulnerable to exploitation. McAfee responds to billions of requests to provide security assessments of IP addresses each day. A complete assessment must be carried out based on the information about the individual IP address itself and any additional information regarding its neighboring IP addresses.

In the security assessment stack, IP reputation is one of the earliest reputations that can be used to quickly identify risk and proactively warn about the potential maliciousness of a download or alert about visiting a web page that may lead to the user’s computer getting infected. Malicious IP addresses, malware hashes, and malicious URLs are the three key indicators of compromise (IoC) in modern cybersecurity defense. Malware infections and advanced persistent threats can be identified when the associated IP addresses are detected as malicious. In addition, phishing attacks can be prevented by blocking suspicious websites associated with malicious IP addresses.

Today, the approach of dirty-listing IP addresses is widely used. For instance, a host sending information to a dirty-listed IP address may be investigated for infection. The drawbacks of dirty-listing are limited scalability as the lists need to be continuously updated when new malicious IP addresses emerge. It is often also a reactive approach as an IP is added to the dirty-list only after it is observed as suspicious. As a result, attackers can easily bypass an IP dirty list by using new IP addresses that have not been employed in malicious activities. The new IP address will often exhibit similar behaviors or present similar IoCs as the original IP, but dirty-listing techniques cannot scale to protect from the growing exploits and zero-day attacks.

Traditional detection techniques look at each protocol and do not accurately account for all protocols served by an IP address. For example, a web reputation might include the reputation for web traffic but does not account for the overall reputation of the entire IP. Likewise, the email reputation can account for email traffic. Ideally, a comprehensive assessment of all services associated with an IP address would give a holistic view of its nature; however, the IP reputation should account for all the neighboring IP address reputations as well. Using graph neural networks (GNNs) to help propagate the IP’s reputation to its neighbors and vice versa can improve holistic knowledge about the IP address and improve accuracy.

There are many challenges to detect malicious IP addresses, including the dynamic nature of IP addresses that are prone to change quickly, the lack of labeled data, and the negative impact of high false positives. Additionally, the voluminous nature of the IPv4 address space is challenging, while the IPv6 address space would be nearly impossible to use in training due to the approximately $3.4 \times 10^{38}$ IP addresses. The nature of the data suggests that an intelligent algorithm for detecting clusters is needed even to be considered feasible. Without proper assessment, over-blocking may occur since IP addresses can provide services for multiple unrelated companies.

To address these challenges, we develop a cross-protocol approach for detecting malicious IP addresses by leveraging metadata from the web, email and domain name system (DNS) protocols using a supervised learning approach. In addition, we build on the first experiment by creating relationships between IP addresses to establish a path for reputations to propagate throughout the graph and have related IPs influence each other’s reputation. We experiment these relationships by connecting IPs using several methods based on subnet-C, subnet-B, and subnet-A membership, connecting by geolocation and autonomous system (AS) memberships and attributes associated with the IP address such as known botnet family membership. By constructing graph edges that reflect such memberships, we can apply graph convolutional networks (GCNs) model to follow the edges and aggregate data from neighbors during training. As a result, we generate predictions that reflect what we know about each IP and the neighbors of those IPs.

Our goal is to perform a large-scale classification of malicious IP addresses that leverage cross-protocol telemetry to produce a context-aware security assessment. The key contributions include (1) performing cross-protocol feature extraction using a supervised learning approach that demonstrates high performance in a real-world data set, (2) constructing a graph of IP addresses, and (3) conducting embedding extraction using GCNs in a semi-supervised manner with only $5\%$ labeled data for classifying malicious IP addresses at scale.

## Related work

This section reviews related works in the area of IP reputation assessment and statistical graphical analytic research. For contrast, our method is compared to other works which have various limitations. Our research draws inspiration from state-of-the-art experiments, builds on the best components transferable to designing a solution for our problem, and solves the limitations of existing technologies.

### IP dirty-listing approach

IP dirty-listing is common in the cybersecurity industry to defend against adversaries. Dirty lists are developed by analyzing various cyber threat intelligence from many resources, including third-party houses. Traditional security approaches apply this methodology to block malicious IP addresses [2–5]. Zhang et al. [6] proposed a page-rank algorithm called highly predictive blacklisting, which ranks adversarial attempts based on the compiled threat intelligence insights. Soldo et al. [7] presented an implicit recommendation system that extended Zhang’s work by examining the temporal patterns of cyber-attacks to rank attack sources. However, these dirty-listing approaches have scalability challenges and are difficult to maintain. Therefore, they are not practical to tackle evolving threats.

### Feature extraction for detecting malicious IPs

Cyber security companies, Internet service providers (ISPs), and cyber law enforcement have researched malicious IPs by isolating commonly shared features among threats. These features can then be detected during incoming network traffic to disposition whether blocking that IP should occur or not. Renjan et al. [8] collected IP metadata (e.g., ASN, ISP, geolocation, user disposition) as features. They combined their data with other reputation data sources and used the new reputation score to classify new IPs. However, they determined that, for malicious IPs, a large quantity of the feature information was unavailable. McGrath et al. [9] proposed several other features (e.g., number of IP addresses with a domain, number of ASs, DNS record TTL) while Moghimi and Varjani [10] proposed a set of even different features (e.g., number of dots in URL, SSL certificate, URL length, dirty-listed keywords for phishing URLs). Bilge et al. [11] proposed a system to detect malicious domains using four different sets of features: time-based features, DNS answered-based features, TTL value-based features, and domain name-based features.

Other researchers developed methods including time-series aggregated data and analytics specific to exposure and blocking malicious activity online [8, 12–18]. For example, Esquivel et al. created a spam-focused reputation system for IPs that send emails by exploring the reverse DNS (RDNS) and sender policy framework (SPF) [12]. They categorized each IP over time to establish a benign or malicious labeled reputation, assessing this reputation efficacy to identify email spam from a real-world dataset. Bajaj et al. [14] concluded that filtering reputation is fundamental to email anti-spam efforts, noting a critical need to create self-learning anti-spam filters combined with automated update procedures.

In contrast to these works, we leveraged cross-protocol telemetry from port 25 for email, ports 80 and 443 for the web, and port 53 for DNS. We combined the features associated with spam, malicious websites and DNS to enhance the signals detecting malicious IP addresses.

### Statistical graph analysis to detect malicious IPs

Some research works have demonstrated that suspicious activities are not uniformly dispersed over the Internet; they often bunch together, forming high-risk ecosystems. Coskun et al. [18] examined Internet traffic between source and destination IPs using a graph database, utilizing weighted edges between connections. They compared IP addresses’ statistical residuals with dirty lists to determine if the larger cluster had a statistical dependency or divergence with the dirty-list data, uncovering previously undetected maliciousness. Moura et al. [19] introduced Internet bad neighborhood and identified high-risk networks that hosted malicious activities. A network was considered high risk if the count of malicious activities exceeded a specified threshold. Collins et al. [20] examined network spatial and temporal data to predict IP address malicious botnets. Stone-Gros et al. [21] authored finding rogue networks, identifying the highest amount of malicious activities via the responsible IPs.

These papers inspired us to leverage the idea that malicious activities tend to be clustered. Therefore, we created graph structures of IP addresses.

### Machine learning to detect malicious IPs

Machine learning (ML) has been applied to detect malicious IP addresses [16, 22, 23]. However, most research focuses on only one protocol (e.g., web, URL) for a targeted use case. In the work of [22], malicious domains and IP addresses were detected based on web data using a graphical approach—a loopy belief propagation algorithm was used to infer each domain and IP reputation. They utilized a real-world data set, assessing the efficacy with 75 M node and 185 M-edge graph. The algorithm served as an in-field tool for initial ML classification and training. Antonakakis et al. [16] used an ML classifier to train DNS record features to establish a reputation. Their model created clusters of real-world DNS records utilizing a decision tree with logit-boost as the reputation function. Their reputation approach categorized, with high accuracy, newly created domains. Huang et al. [23] proposed a cross-protocol method to leverage multiple signals to enhance the detection of malicious IP addresses using supervised learning. In our work, we extend the traditional ML approach to build a graph-based detection system that will scale and be dynamic with only a small portion of labeled data.

### Graph neural networks

GNNs were initially introduced by Scarselli et al. [24], which built on neural networks (NNs) representing process data in graph form. Several survey papers provided an overview of GNN algorithms and applications. Wu et al. [25] work described a wide range of GNN applications and an in-depth synopsis, including GCNs, graph attention networks, graph autoencoders, graph generative networks, and graph spatial-temporal networks. Zhou et al. [26] explored variants and their applications of GNN models, notably spectral methods, non-spectral methods, graph attention networks, and gated GNNs while categorizing by graph, propagation, and training types.

GNNs have received growing attention recently, and have been well-explored, including computer vision, text mining, molecular structure, physical systems, and knowledge graphs [25–29]. Some interesting applications include an anti-abuse detection system at YouTube [30], Covid-19 pandemic forecasting [31], and social networks [32]. In more recent work, Halcrow et al. [30] designed a new approach for addressing the problem of constructing large-scale graphs with sparse data for optimal semi-supervised learning. Targeted towards big data, Halcrow’s method solves the scalability challenge by employing locality sensitive hashing techniques to reduce the number of edges that require scoring. It learns a task specific model and builds a high-performing nearest neighbor graph. This methodology is widely used at Google, especially for detecting abuse on YouTube. It also could be a good application for building graphs that detect exploits on the Internet where labeled data is very sparse. During the global Covid-19 pandemic GNNs [31] were used in a novel forecasting approach. Widely available temporal data was enriched with spatial data like inter-region interactions allowing the model to learn complex dynamics. To accurately capture the spatial interactions the researchers used available GPS data collected from mobile devices. The resulting algorithm was used as a powerful tool to understand the spread and evolution of the virus during the pandemic. Epasto et al. [32] introduced an unsupervised graph embedding method called Splitter which allows graph nodes to have multiple embedding to increase encoding of their multi-community membership (i.e., the social network users). The application of this work can be extremely useful in link prediction, visual discovery, and exploration of the multi-community node membership.

In addition, DeepWalk [33] learns hidden social representations of vertices in a network where information from truncated random walks is input to learn representations that encode structural regularities. The method has been demonstrated to be effective in challenging multi-label network classification tasks. Message passing neural networks can be applied to molecular graphs in chemical compounds to predict quantum properties of organic molecules [34]. Another application is using GNNs to predict a patient’s risk of cancer. In this case, there is typically a very small quantity of labeled data, so a skip-gram solution was proposed to leverage edge features to infer patient similarity [35].

Based on the real-world challenges, many researchers have investigated how to improve GNN performance for large-scale data. One negative side effect of using graphs is that performance can degrade significantly as graph size or the amount of relationships within the graph increase. To improve performance, Page Rank can be computed prior to training such that it does not need to be calculated on each layer saving valuable computational cycles [36].

One possible negative side effect of combining node features and connections information is a new bias. Metadata orthogonal node embedding training (MONET) removes bias by training embeddings on a hyper-plane orthogonal to that of the node features. Bias is reduced by partitioning the graph to provide the ability to interpret results and separating the topology embeddings from metadata embeddings [37].

Despite numerous types of GNNs, GCNs are an emerging field. Zhang et al. [38] provides an exhaustive survey on GCNs, including spectral-based and spatial-based models. These models depend on convolution type and their applications (e.g., computer vision, natural language processing, and social networks). A number of pioneering works generalized well-established convolution neural networks on arbitrarily structured graphs [39–42]. Inspired by [39, 41], Kipf and Welling [42] proposed a simplified GNN model, called a GCN, whose graph datasets achieved exceptional performance for classification. GCNs were applied to semi-supervised learning on node classification [42], graph classification [43], and link prediction [44]. In this work, we used GCNs for semi-supervised learning on node classification to detect malicious IP addresses.

GNNs have received growing attention and have been explored in a wide range of applications, including computer vision, text mining, molecular structure, and social networks. We apply GCNs to address IP reputation assessment, a novel application within the critical domain of cybersecurity.

**Fig. 1:**
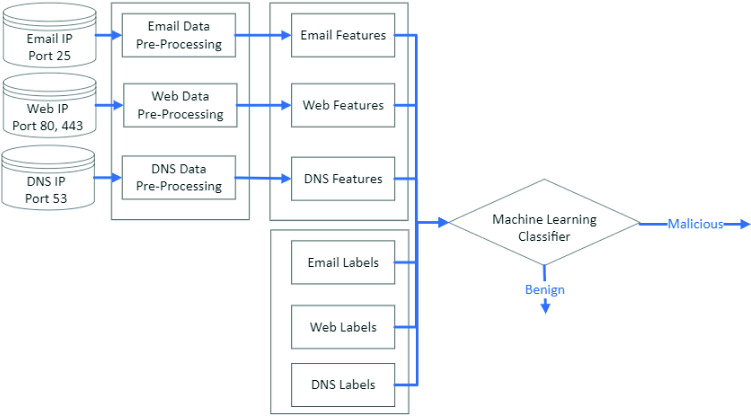
Framework for the cross-protocols analysis

## The cross-protocol analysis method

### Data collection

In the first phase of our research, we developed a cross-protocol scheme to detect malicious IP addresses (Fig. 1). The scheme incorporated a typical machine learning pipeline: a collection of personal identifiable information (PII) -protected data from applicable ports, pre-processing of email, web and DNS data, feature engineering, labeling of the records, classification, and finally evaluation.

Data was collected via telemetry from port 25 (email IP addresses), port 80 and 443 (web IP addresses), and port 53 (DNS IP addresses). There were two datasets as described in Table 1. The first dataset consisted of email and web data. It had 57,494 samples (40,347 benign, 17,147 malicious ). The feature set had 125 email features and 117 web features. The second dataset consisted email, web and DNS data. It had 200,085 samples (183,539 benign and 16,546 malicious). The feature set had 100 email features, 95 web features and 50 DNS features.

**Table 1:** Datasets

Dataset	Sample size	Features	Feature size
1	57,494	Email, web	242
2	200,085	Email, web, DNS	245

### Feature extraction

In the feature extraction phase, we targeted the derivation of the highest relevant and most effective features, which also had properties of extraction simplicity, transformation invariance, and contributed to the segregation of malicious and benign IP addresses. Tables 2, 3, and 4 depict, respectively, a sampling of web-derived, email-derived features and DNS-derived features. As described in Table 1, in our first dataset, we identified a set of 242 features derived from email and web IP addresses. In our second dataset, we identified a set of 245 features from email, web and DNS IP addresses. Asserting the most applicable features was also a building block for further experimentation with GCNs.

**Table 2:** Sampling of email IP features

Email IP features	
‘d_g_msg_rate’	
‘d_mo_msg_rate’	
‘d_msg_count’	
‘d_spam_count’	
‘d_wk_msg_rate’	
‘mo_msg_count’	
‘newsender’	
‘wk_msg_count’	
‘rate_d_msg_count’	
‘rate_mo_msg_count’	
‘rate_wk_msg_count’	
‘wk_g_msg_rate’	
‘wk_mo_msg_rate’

**Table 3:** Sampling of web features

Web IP features	
‘alexaRanking’	
‘associatedIpCount’	
‘associatedIpData’	
‘associatedReputationSpam’	
‘associatedReputationSpamAvg’	
‘associatedReputationSpamMax’	
‘catserverCategoryExactMatch’	
‘catserverReputationExactMatch’

**Table 4:** Sampling of DNS IP features

DNS IP features	
‘verynewdns’	
‘newdns’	
‘newishdns’	
‘source_change60’	
‘source_change30’	
‘source_change7’	
‘d7cmax’	
‘d7cmin’	

**Fig. 2:**
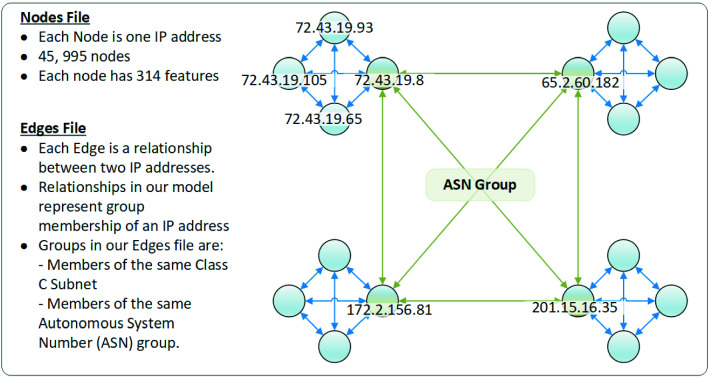
IP addresses graph structure with IPs as nodes and ASN and Class C-subnet groups as edges

### Random forest classification

The cross-protocol solution employs a random forest (RF) algorithm [45]. As most ML models do, its performance depends on data that embodies high-quality labels and appropriate features to optimize a predictive output. Our study ensured high-quality labels and features that are relevant and effective to take advantage of multi-protocol IP sources. We obtained connected samples for email, web and DNS IP addresses in the data collection phase. After data pre-processing and applicable labeling, the total sample size for the first dataset was 57,494 (40,347 benign, 17,147 malicious ) and the total sample size for the second datasset was 200,085 (183,539 benign and 16,546 malicious). The data were randomly split 70/10/20 for training, validation and testing sets. During a training process, the forest was built with numerous trees with randomly sampled features as input, applying the same distribution for any tree in the forest and controlling variance. The resulting prediction was determined by majority or weighted voting. There are many advantages of RF, which motivated us to apply it to our solution. Some of them include resistance to over-fitting, a low number of control and model parameters, feature elimination during training, ability to handle a large number of features, robustness to noise and outliers, and most importantly, the model’s variance decreases as the number of trees increases, but the bias remains steady.

### Random forest evaluation

The evaluation metrics for our study included: root mean square error (RMSE), receiver operating characteristic (ROC) curve, F1-score, precision, recall, and area under the curve (AUC) in the ROC. The “positive” means malicious IP and “negative” means benign IP.

## The graph neural network method

### Graph construction

In researching security threats, we know that IP addresses that exhibit similar malicious behavior are often related to each other in some manner. Data collection of IP behavior can document suspicious and malicious behavior but does not reflect the relationships that can better inform us.

To represent relationships between IP addresses, in the second phase of our research, we constructed a network graph for IP data (Fig. 2). IP addresses are the nodes, and IP connections are the edges. The IP address dataset consists of binary classes of IP metadata. The number of IP addresses is *N*, and the feature dimension is *D*, where $$N = 45{,}995$$ and $$D = 242$$. The class label is $$C= 0$$ (benign) and $$C= 1$$ (malicious). The total edge number $$ E = 112{,}182$$.

The input to the GCN model was created in two parts. The first part was an input file for the nodes (or vertices) of the graph network, named node file. The second part was an input file for the edges between the nodes, named edge file. We constructed the nodes and edges as follows. The node file consisted of node and associated features for each node. Each line in this file was a single data point, an IP address. The first column was the IP address, followed by 242 features and the last column is the label for the node. The edge file consisted of two connected nodes for that edge.

$$\begin{aligned} \begin{array}{l} \text {Node File:} \\<IP\_address>
<meta\_data>
<class\_label>\\ \text {Edge File:} \\<IP\_i>
<IP\_j>\ \text {if IP}\ i\ \text {has connection to IP}\ j .\\ \end{array} \end{aligned}$$

Convolution filters are designed to examine sub-graphs of the larger graph to recognize patterns better. Furthermore, in a fully representational network graph, the intermediate entities (e.g., countries, ASNs, etc.) do not generate feature data because, in practice, those intermediate entities are not endpoints themselves. Because they are not endpoints, intermediate entities have no behavior data to record.

A convolution model is configured to look at the surrounding neighborhood of a data point during training. The nodes that are directly connected are the first-order neighbors in the graph, the second-order are the nodes that are neighbors of those neighbors. The GCN model weights the feature information of the node itself first, then the first-order neighbors at a reduced weight, and then the second-order neighbors at a further reduced weight.

The first convolution cycle had no feature information for the first-order neighbors when we attempted to utilize intermediate entities as nodes in our network graph. Although second-order neighbors contributed feature information for training, the contribution was so weakened that the outcome had poor training results. The resulting convolutions were as follows:

$$\begin{aligned} \begin{array}{l} \text {Original node (IP address, 242 features)} \\<-> \text {1st-order neighbor (intermediate entity, zero features)} \\ <-> \text {2nd-order neighbor (IP address, 242 features)}.\\ \end{array} \end{aligned}$$

Rather than including featureless intermediate entities as nodes in our model, we removed them, leaving only the IP addresses themselves. We then constructed direct edges between IP addresses that had relationships because they belonged to the same groups (e.g., country, ASN etc.). The resulting convolutions were as follows:

$$\begin{aligned} \begin{array}{l} \text {Original node (IP address, 242 features)} \\<->\ \text {1st-order neighbor (IP address, 242 features)} \\ <-> \text {2nd-order neighbor (IP address, 242 features)}.\\ \end{array} \end{aligned}$$

We ensured that each convolution had valid feature information for computation using this approach. This further resulted in improved accuracy after training. It also confirmed two initial conclusions for using a GCN model. First, we needed to remove intermediate entities from the node input file, leaving only IP addresses. Second, we needed to construct direct IP to IP edges for those nodes when they shared a relationship.

**Fig. 3:**
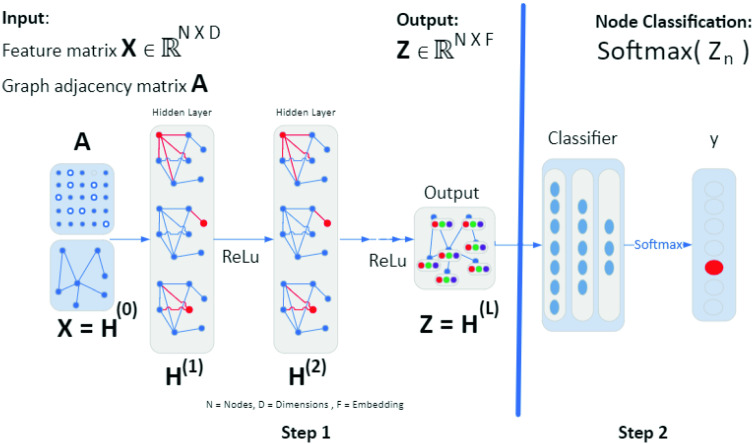
Graph-based semi-supervised learning framework using GCN model on node classification for IP reputation

The next inquiry for the construction of our GCN was to determine what kind of edges to include in our edge input file. We answered this question by first constructing a series of edge lists based on different relationship types, and then testing each edge list individually, and in combination. Another constraint we encountered was the size of the edge list and its negative impact on computation time. In fact, the size of the edge list eventually exceeded the memory capacity of our hardware platform. We then explored ways to reduce the size of our edge list during training experiments.

### Semi-supervised node classification using GCNs

We selected the GCNs developed by Kipf and Welling [42]. The model encoded both the graph structure and node features, in a semi-supervised training structure. For a GCN model, *f*(*X*, *A*), $X\in {\mathbb {R}}^{N\times {D}}$ is a input feature matrix, where *N* is sample size and *D* is feature dimension. *A* is a graph adjacency matrix with added self-connections, where $\tilde{A}=A+I_N$ and $I_N$ is the identity matrix. In GCN model, a convolution operation inputs the node information for itself (i.e., a self-loop), as well as the node information of its immediate neighbors. Mathematical operations on such irregular input data results in exploding or vanishing gradients, resulting in numerical instability and unsatisfactory results [42]. To properly handle this instability, GCNs introduced a re-normalizing trick, utilizing a simplified Chebyshev approximation technique [46], with the following equation.

1 $$\begin{aligned} Z=\tilde{D}^{\left( -\frac{1}{2}\right) }\tilde{A} \tilde{D}^{\left( -\frac{1}{2}\right) }X{\varTheta }, \end{aligned}$$

where ${\varTheta }\in {\mathbb {R}}^{D\times {F}}$is now a matrix of filter parameter, *F* is filters and feature maps, $Z\in {\mathbb {R}} ^{N\times {F}}$ is the convolved signal matrix, and $\tilde{D}_{ii}=\sum {\tilde{A}_{ii}}$.

In Fig. 3 the local structure of the network graph is represented in this equation by the adjacency matrix *A*. The adjacency matrix records which nodes share an edge with the input node, leading to the computation of the node information for all those neighbors. As part of the Chebyshev approximation, this equation introduces the node identity to the adjacency matrix, effectively including the information of the node itself (i.e., a self-loop) into the computation of node features. In order to regulate the output of the node information, GCN utilizes a re-normalization trick which multiples the results of the node computations by the “inverse reciprocal exponent” of the node degree. The node degree is defined by the number of edges a node has; thus, a node with a larger number of edges will have a higher degree, and the computation of more node information is multiplied by the reciprocal inverse exponent of the degree number. By doing this the influence of an adjacent node is reduced according to how many other adjacent nodes are in that convolution.

After aggregating and normalizing the feature information from the node neighborhood, the information is put into the neural networks. Our model was configured for two layers. Each layer consisted of 16 neurons. At each layer, the parameters were shared for all nodes. Because the underlying data is non-Euclidean, the preferred method was to activate the neurons in a way that was appropriate for complex data. Rectified linear unit (ReLU) activation was used to process the aggregated node neighborhood representations through the neural network and continue training. After activation, each hidden layer established hidden weight matrices and sent the data forward to an output layer. Between the convolution layers, dropout was introduced. The dropout both reduced the possibility of overfitting and conserved computational resources.

The problem of GCN node classification of IP addresses can be framed as graph-based semi-supervised learning. The label information was smoothed over the graph via graph-based regularization using a graph Laplacian regularization term in the loss function. The assumption was that the connected nodes in the graph were likely to share the same label. In this work, we encoded the graph structure directly using a model *f*(*X*, *A*), and trained on a supervised target loss for all nodes with labels. The adjacent matrix *A* of the graph allowed the model to distribute gradient from the supervised loss and enabled the model to learn representations of both types of nodes, with and without labels.

There are two steps in node classification as shown in Fig. 3. The first step is to automatically extract the embedding using a GCN model. The objective is to learn a function of features on a graph. The main idea is to pass messages along the edges of graph, agglomerate and transform. The input was feature matrix $X\in {\mathbb {R}}^{NxD}$ and adjacency matrix $A\in {\mathbb {R}}^{NxN}$. The feature matrix consists of input features for nodes. *N* is the sample size and *D* is the feature dimension. The adjacency matrix *A* is the representation of graph structure. It uses binary values 0 or 1 which represent ‘no connection’ or ‘has connection’ between node *i* and node *j*. The neural network hidden layers perform layer-wise propagation $H^{l+1}=f(H^{l},A)$. The output is a feature matrix $Z\in {\mathbb {R}}^{DxF}$. The feature matrix is the embedding of the output features for nodes. *F* is the feature embedding dimension. The second step is to perform classification and map to probabilities for each node using the softmax function.

In this work, a GCN model is applied to the large-scale network graph, consisting of 45,995 nodes and 112,182 edges. The nodes are IP addresses, each of which may have only a few neighbors or hundreds of neighbors. Figure 3 shows the GCN network architecture for node classification. The input consists of feature matrix *X* and adjacency matrix *A*. A hidden layer encapsulates each node’s representation by aggregating feature information from its neighbors. After feature aggregation, a nonlinear transformation ReLu is applied to the resultant outputs. We use Adam optimizer and Chebyshev polynomials basis filters with polynomial order $= 4$. The output layer from the first step was *Z*. The second step is to perform node classification. The final output is a probability score for each node belonging to the target class.

**Fig. 4:**
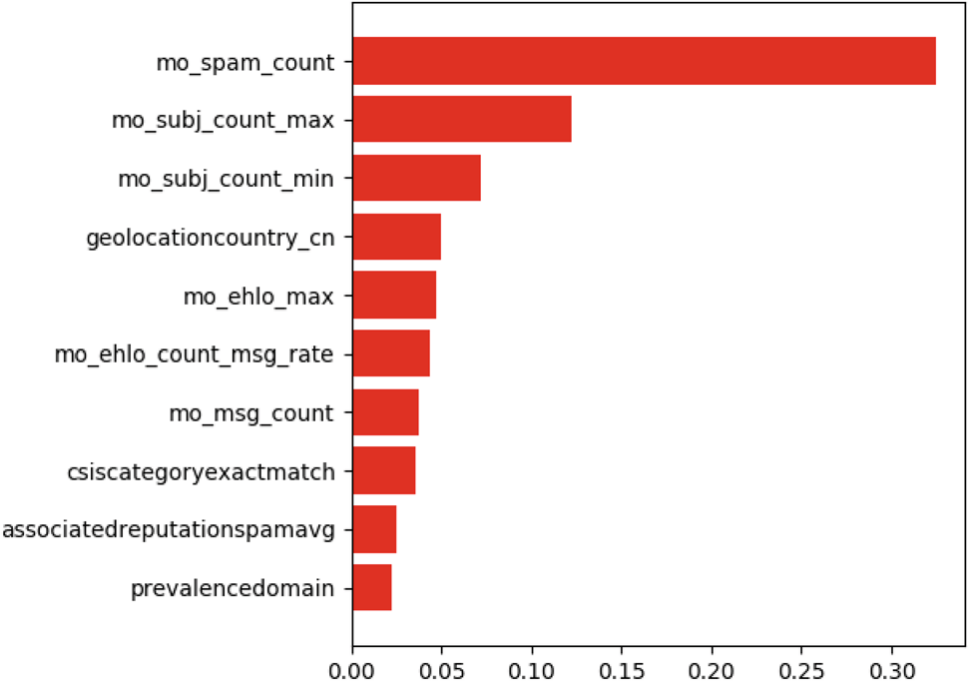
Feature importance for the top 10 features

**Fig. 5:**
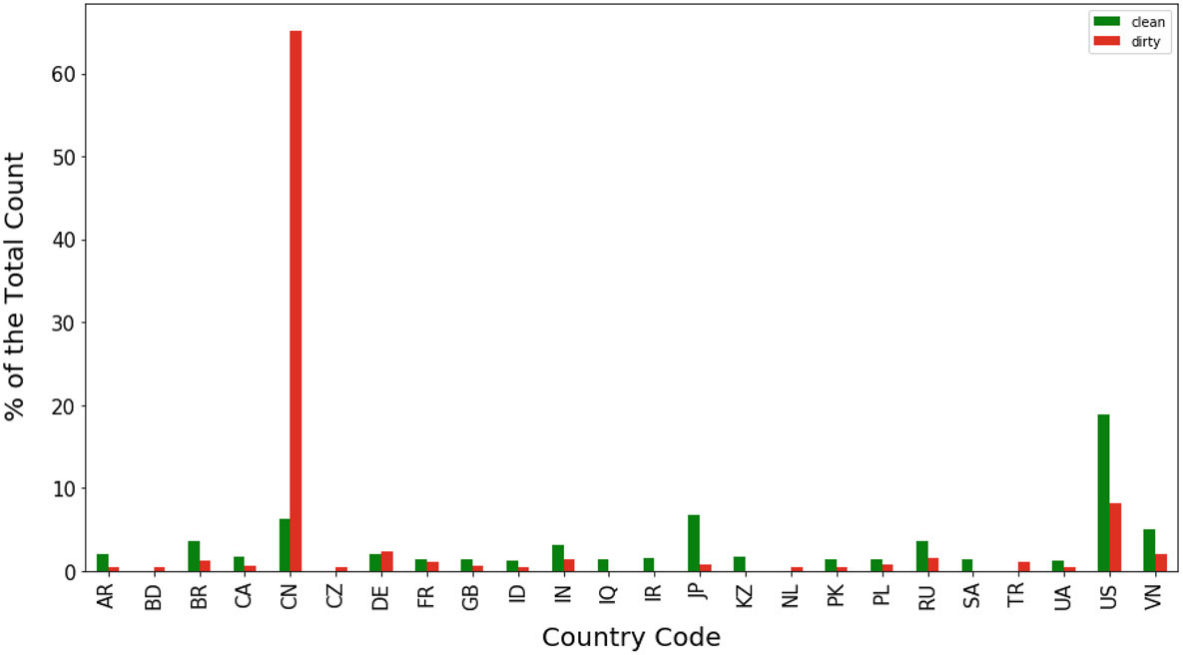
Histogram of country code

### GCN evaluation

We experimented with several GCN hyperparameters. We also performed experiments by making changes to both the node and edge files of the graph network. We focused on a sample of the data, as doing an exhaustive IP to ASN mapping with all the web and email metadata is too large of a dataset to realistically process. To evaluate the relative performance of these experiments, we computed both an accuracy score and a cross-entropy loss score. We arrived at the optimal combination of hyper-parameters and graph network configuration using these scores.

## Experimental results

### Cross-protocol analysis

The cross-protocol analysis was performed using connected email, web and DNS data that was sampled from McAfee network traffic. We conducted two experiments on cross-protocol analysis based on the two datasets as described in Table 1. The first experiment used email and web features. The second experiment used email, web and DNS features. In paper [23], the classification results of the first experiment were presented. We only reported the classification results of cross-protocol analysis for the second experiment in “Classification results of cross-protocol analysis”.

**Table 5:** Random forest testing results

Experiment	Feature size	AUC	F1-score	RMSE
Email & Web & DNS	245	0.9896	0.9773	0.0614
Email only	100	0.9748	0.9312	0.1081
Web only	95	0.6647	0.2233	0.6944
DNS only	50	0.5140	0.1561	0.9441

#### Feature analysis in cross-protocol analysis

We visualized the feature importance for the model and drew a histogram of the top features for the combination of malicious and benign samples. The feature importance was computed based on Gini importance or mean decrease impurity from the random forest structure. Figure 4 shows the feature importance of the top ten features based on training on the first dataset with email and web and features. As an example, we took ’geolocationcountry_cn’ and visualized it on a histogram in Fig. 5. The feature has a clear discriminant power for distinguishing malicious and benign IP samples because high density of dirty appears in ’geolocationcountry_cn’ compared to other geolocations. Even though other geolocations were represented in the data, none were identified as important features. Security researchers confirmed that the feature importance was valid.

#### Classification results of cross-protocol analysis

We performed four experiments. First, we trained three RF models using only *email*, only *web* and only *DNS* features. Second, we trained a model using a combination of *email, web and DNS* features. Table 5 shows the test performance metrics for the four experiments in terms of the AUC, F1-score, and RMSE. The model incorporating cross-protocol features has the highest AUC, the highest F1-score, and the lowest RMSE compared to models with only one protocol.

**Fig. 6:**
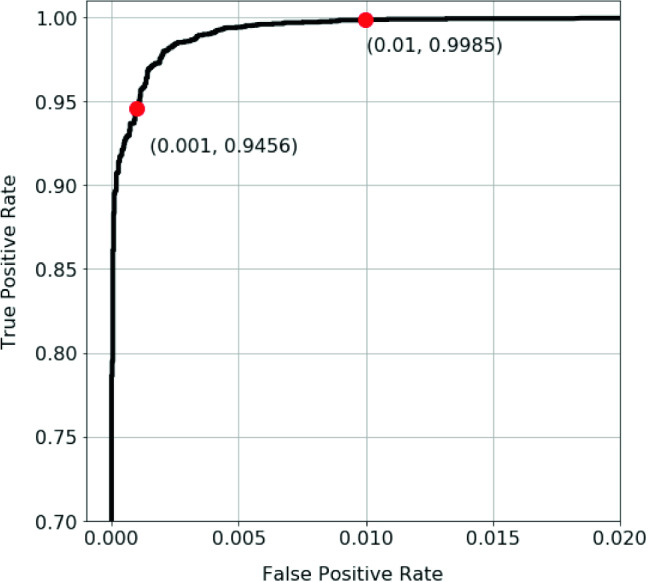
RF test set ROC for email & web & DNS data

Figure 6 shows the RF classifier test ROC for email & web & DNS data. The two operating points, which are highlighted in red in the curve, are associated with two sensitivity levels. The model achieved a detection rate of TPR/$\hbox {recall} = 94.56\%$ at $\hbox {FPR} = 0.1\%$ with $\text {precision score}=98.85\%$ and $\text {F1 score}=96.66\%$. With a higher tolerance for falses of $\text {FPR} = 1.0\%$ the model achieved TPR/$\text {recall} =99.85\%$ with $\text {precision score}=89.68\%$ and $\text {F1 score}=94.49\%$.

**Fig. 7:**
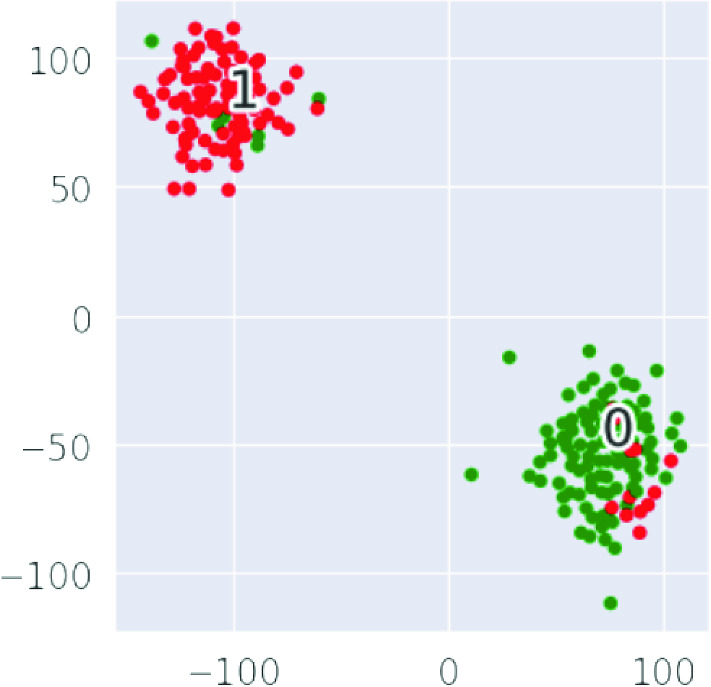
The t-SNE visualization of the features in the test set (0 = benign, malicious)

To further demonstrate test data and test classification results, we used the t-SNE visualization of the features for $\hbox {perplexity}=75$, $\hbox {iteration}=3000$, and $\text {learning rate}=700$ as shown in Fig. 7. The plot shows the top 100 malicious IPs (red dots) and the top 100 benign IPs (green dots) in the test set. A clear separation of the malicious IPs (1) and benign IPs (0) for the test set can be seen. A few samples that are not well separated show classification errors.

### GCN results

The graph neural network analysis was performed using connected email and web data. We conducted the GCN experiments on the first dataset as described in Table 1 using email and web features. To fit the memory limit, we randomly selected $80\%$ data to construct the graph with 45, 995 nodes, where 13, 753 were malicious and 32, 242 were benign.

**Table 6:** Test results for different graph structures and filters

Node #	Edge #	GCN filter	Edge	Accuracy
45,995	222,264	Local pool	Sub-C, botnet	0.6991
45,995	222,264	Chebyshev	Sub-C, botnet	0.8559
45,995	1,722,108	Chebyshev	Sub-C, botnet	Memory error
45,995	112,182	Chebyshev	Sub-C, ASN	0.8952

**Table 7:** Test results on GCN

GCN filter	Edge	Accuracy	Precision	Recall	F1 score
Chebyshev	Sub-C, botnet	0.8528	0.8890	0.5803	0.9022

#### Graph structure experimentation and GCN optimization

In our first experiments with the GCN model, we constructed a graph for IPs by creating relationships between IP addresses by linking them by Subnets (Class C, B, and A), ASNs, geopolitical codes, and, if the data existed by botnet identification. However, not having any restrictions on the graph led to poor results. After further investigation, it was determined that IPs identified as “non-endpoints” were not assigned to individual hosts and lacked behavioral features; thus, they did not contribute during training. Therefore, we removed the sparse data to solve this problem and built a final “nodes file” of 45, 995 IPs.

Next, we experimented with the type and degree of edges used to construct the graph. In our data set, we related IPs in one of four ways. In the first relationship type, an IP belongs to a relationship of ASN, which is the most extensive inclusion set of IPs. In the second relationship type, we also related the IPs based on class C-subnet in the second type of relationship. All class C-subnet relations exist within the same ASN, but an ASN contains multiple class C-subnets. All class C-subnets typically reside within approximate geolocation. In the third relationship, we used the geolocation of the IP address. In the fourth relationship, we used a security feature to group IPs based on the same botnet family participation. We made a separate relationship edge list for each type of relationship to test them individually and then subsequently test each relationship in combination. One drawback to this approach is that the relationship combinations can produce massive data sets, which cause memory errors when training. To resolve this problem, we applied a solution of data reduction. Running experiments on graph construction, we concluded that no matter which network structure we tried, class C-subnet had to be an essential part of the graph to achieve ultimate results. One explanation could be that IPs that are close numerically are more likely to be part of the same subnet and, therefore, exhibit similar behavior.

In addition, we reviewed GCN hyper parameters while training. The parameter that provided the most significant impact was the pooling operation. Specifically, the Chebyshev filter produced better accuracy.

#### GCN classification results on different graph structures

The summary of the results for Graph structure and GCN hyperparameter selection is shown in Table 6. We conducted all the combinations of different graph structures with dozens of experiments. We only report four results here based on page limitations. We split training, validation and test sets into $15\%$, $15\%$ and $70\%$. There were 6900 nodes for training, 6900 nodes for validation, 32, 195 for test. Training the GCN model on the feature-rich data set with local pool filter and sub-C botnet edges produced a baseline accuracy of 0.6991. With the same edge setting, the Chebyshev filter produced better accuracy of 0.8559. Training the full set of edges with the relationship combinations caused a memory error.

We achieved the highest accuracy when we combined a reduced data set using the Subnet-C relation with ASN relations. Training the model on a complete set of IPs resulted in memory exhaustion errors; therefore, we reduced the ASN data set to 30 percent of the edges. By further reducing the ASN data set to two percent, we discovered it would decrease the accuracy score by only one-tenth of a percent. The accuracy was 0.8952 under the conditions of Chebyshev filter and Subnet-C relation with ASN and half-edge number.

#### GCN classification with small number of labeled data

After we selected the graph structure and GCN filter type, we conducted experiments to compare the test performance with a different number of labeled training data. In the experiments, we used the graph structure of 45, 995 nodes and 112, 182 edges under the conditions of Chebyshev filter and Subnet-C relation with ASN Table 7. For the 45, 995 nodes, 13, 753 were malicious and 32, 242 were benign.

The goal of this experiment is to verify the GCN classification performance with only a small number of labeled nodes. To produce our results, we randomly selected $5\%$ training, $5\%$ validation, and $90\%$ test data. There were 2299 nodes for training, 2299 nodes for validation, 41, 397 for test. The accuracy on the test set achieved was 0.8528 with a precision of 0.8890, recall of 0.5803, and F1-score of 0.9022 as shown in Table 7. To reiterate, that was training on only $5\%$ labeled data. This result is promising and highlights the ability of using a graph-based semi-supervised approach to detect malicious IP addresses. We re-ran the experiments with different ratios of training, test, and validation and achieved similar results.

## Conclusion and future work

This paper demonstrated a two-phased approach to developing a scalable graph-based IP security assessment. In the first phase, we built a Random Forest model with an innovative approach towards features used to identify malicious IP addresses. Our cross-protocol approach combined targeted features from email and web protocols.

We further tackled the problem of detecting malicious IP addresses at scale, where only a small subset of labels was available. We framed the challenge as a graph-based semi-supervised learning problem. The optimal feature set from the cross-protocol assessment was used as node features in the graph. As far as we have researched, this innovative approach of combining traditional machine learning with semi-supervised graph-based detection for malicious IPs has never been attempted.

In conclusion, traditional IP filters that are applied to single protocol reputation systems are not sufficient to protect against malicious activity in the current threat landscape. A combination of protocol feature vectors can be used by connecting the related IP addresses into a graph network to improve context and increase accuracy compared to non-connected feature vectors. Given a set of nodes (IPs), node features, and edges between nodes (connected IP addresses), a GCN model follows the provided edges and aggregates data from neighbors during training. Such a system produces predictions that reflect what we know about each IP and what we know about the neighbors of those IPs. Our final model achieved an accuracy of 0.8528 and F1 score of 0.9022 with training on only $5\%$ labeled data. The innovation of adding knowledge about relationships, in the form of graph edges has demonstrated to improve context awareness for Internet security.

In the future, we will explore more edge categories and use weighted edges in training to enhance feature expressiveness. To accommodate even larger data sets, we will also explore approximation methods, such as mini-batch strategies. In addition, we will explore inductive learning on graphs (opposed to our current transductive learning GCN framework), such as GraphSAGE [47], to tackle the challenge of dynamic IP graphs.
